# Looking in the Mouth for Noninvasive Gene Expression-Based Methods to Detect Oral, Oropharyngeal, and Systemic Cancer

**DOI:** 10.5402/2012/931301

**Published:** 2012-09-24

**Authors:** Guy R. Adami, Alexander J. Adami

**Affiliations:** ^1^Department of Oral Medicine and Diagnostic Sciences, Center for Molecular Biology of Oral Diseases, University of Illinois at Chicago, Chicago, IL 60612, USA; ^2^Department of Immunology, University of Connecticut Health Center, Farmington, CT 06030, USA

## Abstract

Noninvasive diagnosis, whether by sampling body fluids, body scans, or other technique, has the potential to simplify early cancer detection. A classic example is Pap smear screening, which has helped to reduce cervical cancer 75% over the last 50 years. No test is error-free; the real concern is sufficient accuracy combined with ease of use. This paper will discuss methods that measure gene expression or epigenetic markers in oral cells or saliva to diagnose oral and pharyngeal cancers, without requiring surgical biopsy. Evidence for lung and other distal cancer detection is also reviewed.

## 1. Introduction

This year half a million people will be diagnosed with oral squamous cell carcinoma (OSCC) or oropharyngeal cancer, and about one quarter of that number will die from the disease, often disfigured from treatment. Cure rates have improved only slightly over the decades. Paradoxically, while early curable lesions are visible in the mouth, they are seldom diagnosed. Because oral and oropharyngeal cancers are often asymptomatic until the final stages, improved screening to detect cancerous lesions in the oral cavity is a key component to reducing this cancer [1]. Detection of suspicious oral lesions by a general dentist includes a visual inspection of the oral mucosa and visible throat. The clinician looks for either nonhomogenous or verrucous surfaces, of unknown cause, whitish or reddish in color. The examination of the oral cavity can be aided by toluidine blue vital staining, because the dye is retained in cells with malignant changes, or by the use of a fluorescent light source as premalignant and malignant lesions may differ from normal mucosa in their production of fluorescence [1]. On the detection of a suspicious lesion (of duration over 2 weeks), the patient may be advised to make an appointment with an oral surgeon so the lesion can be biopsied. The histopathological examination of a stained tissue section by a pathologist is the gold standard for diagnosis of oral neoplasia. A diagnosis is made based on changes in cell and nuclear size and mucosal architecture. Problems with this procedure include the difficulty for all but the most experienced practitioners to know which part of a heterogeneous lesion should be sampled, the need for multiple biopsies, the preference of only some dentists and physicians to perform oral biopsies, the refusal of some patients to submit to oral biopsies, and the subjective nature of the pathological analysis. The invasive nature and skill required to perform the procedure limit its usefulness as a part of oral cancer screening. 

 The procedure of using a cytology brush to harvest mucosal cells from suspicious lesions to detect OSCC and oropharyngeal cancer was first attempted many years ago as reviewed earlier [2]. These cells can be used for cytological evaluation after staining. This methodology, offered by one company in particular, OralCDx, has been promoted over the last 12 years as a substitute for tissue pathology [3, 4]. More ambitious approaches use cells from the oral cavity or throat obtained with a brush or oral rinse as a source of DNA or RNA that can be used to determine mutations [5, 6] or changes in gene expression linked to cancer. Finally, saliva itself can be analyzed to detect RNA that is associated with not only head and neck squamous cell carcinoma (HNSCC) but also systemic cancers. All these approaches offer the admirable goal of eliminating the need for oral biopsy as a methodology of OSCC detection.

## 2. RNA from Brush Oral Cytology

Until 2004 it was largely thought that RNA harvested from the oral mucosa using a cytology brush would be so severely degraded as to disallow the identification of specific mRNAs [7]. Early pilot studies demonstrated that the isolation of RNA from brush oral cytology was possible and that mRNA could be detected using RT-PCR or microarray analysis, but it was not clear how reliable the method was and what was being measured [8, 9]. Remarkably, in their pioneering human study Spivack et al. saw a qualitative correlation of detectable expression of a number of mRNAs in laser microdissected lung tissue and brush cytology cells from the same patients [9]. However, large interpatient variability in mRNA quantitation was seen (up to 10,000 fold), and the source of this variation was not explored. In a pilot study, increased levels of the L5gamma2 mRNA in RNA from brush cytology were found in human oral carcinoma versus normal tissue, though this study lacked statistical analysis of the data [10]. A source of variation in these studies may be methods of RNA preparation and analysis and statistical data evaluation. Finally, pilot studies demonstrated increases in KRT17 mRNA and the splice variant ATP6V1C mRNA in RNA from brush cytology of OSCC [11, 12]. Studies done comparing RNA from brush cytology of benign mucosa of smokers and nonsmokers have shown differential expression of genes by global gene expression analysis that were verified using RT-PCR, though the RNA was not 100% intact [13, 14]. It has been our finding that careful selection of primer target size allows reproducible measurement of specific mRNAs in RNA from brush cytology [15]. Over the last 4 years we have shown that not only is the RNA of high enough quality to allow the measurement of specific mRNAs but also that samples taken over consecutive weeks from OSCC in hamster showed consistent levels of specific mRNAs [15]. Reproducibility was further verified in healthy mucosa in human subjects [16]. 

The difficulty in extracting totally intact mRNA from brush oral cytology is thought to be due to the high level of ribonuclease in saliva present during cell collection, storage, and processing, which results in differences in levels of specific mRNAs [17, 18]. Another important contribution to the RNA quality may be the dead and dying cells that make up the squamous epithelium. In fact a case could be made that brush cytology samples of any squamous epithelium, such as skin, cervix, or oral and pharyngeal tissue, are going to include partially degraded RNA. Despite this, RNA from brush cytology has shown changes in gene expression supported by work from different laboratories when RT-PCR is used with suitable mRNA controls and consistent primer design [15, 16, 19, 20].

There is a large legacy of over 20 earlier studies on global gene expression changes measured in surgically obtained tissues for HNSCC, mainly of the oral cavity, oropharynx, hypopharynx, and larynx [21–25]. These supply a list of over 100 genes that show expression changes correlated to HNSCC that can be tested using RT-PCR with RNA from brush oral cytology of suspect malignant lesions. RNA from brush oral cytology of the oral mucosa represents almost exclusively cells of a single type in contrast to tissue from surgical samples which include variable amounts of stromal tissue. Despite the claims of some brush producers, the brush collects cells from at best the top 2/3 of the mucosal in normal tissue, typically 100% epithelial cells [26]. As is now well understood, analysis of homogeneous cells allows much more sensitive detection of gene expression changes of that cell type [15, 27]. Brush cytology provides nearly pure epithelial cells without the need for tissue microdissection. Work from our laboratory exploited the list of OSCC marker genes in surgically obtained head and neck tumor tissue [21–25]. Twenty-one mRNAs from this list were quantified in RNA from brush cytology samples of OSCC associated with tobacco usage and benign oral lesions. Six mRNAs showed differential expression, and an OSCC classifier was developed based on the level of 5 mRNAs, with 4 from the list (data not shown). These data and results from other laboratories, while requiring further validation, lend support to the idea that a well-designed screen of RNA from brush cytology has the potential to aid in OSCC screening [11, 12, 15, 16].

## 3. RNA from Cell-Free Saliva

While basic research scientists catalogue gene expression, identifying genes actively synthesizing mRNAs that are translated into protein, the clinician is more interested in mRNAs as markers whose levels correlate with disease. For some years efforts have been made to correlate the presence of oral cancer with changes in specific RNAs in saliva [28, 29]. From the start, this approach has been championed by one group that has worked to perform the difficult task of cataloging mRNA fragments in cell-free saliva. With the rationale that the best screening method uses the easiest sample collection, the Wong group has kept the methodology simple. No effort is made to induce salivation; patients are only asked to refrain from eating one hour prior to sample acquisition and samples are taken at a uniform time of day and placed in a RNA preservative. 

The original finding that RNA could be detected in saliva was unexpected despite the fact that multiple RNA forms had been detected in plasma [30]. It was originally thought that no RNA could survive extracellularly due to the high level of ribonuclease in saliva [17, 18, 31]. Although one report showed RNA is undetectable in saliva, there is ample evidence that there is extracellular RNA in saliva at very low levels [32–34]. The source was originally thought to be nearby lysed oral or pharyngeal tumor cells. However, measured changes in salivary RNAs with systemic disease, such as mammary and pancreatic cancer, led to the idea that RNA in saliva comes from plasma prior to its secretion by the salivary glands [35, 36]. Now it is understood that while RNA can be found in plasma or saliva, perhaps bound to proteins, another source is exosomes [37–39]. These small membrane-bound vesicles can be produced in many cells and are thought to be part of a not well-understood exchange system for mRNA, miRNA, protein, and other macromolecules. Because exosomes are thought to freely travel in the blood and possibly exchange in other body fluids such as saliva, it would then be understandable how diseases at distal sites can contribute to saliva RNA populations. Also, perhaps with the acceptance that most saliva mRNAs are likely to be in exosomes, appropriate refinements in RNA purification may improve quality and yield of saliva RNA preparations [39].

 The evidence for saliva-based RNA as a classifier for OSCC, and that changes in saliva RNA levels are linked to a variety of diseases, is almost exclusively from one laboratory. On the subject of OSCC there is a published study from another group that showed RT-PCR detectable MMP1 in saliva in 20% of OSCC patients but not in any healthy controls [40]. In this study RNA from centrifuged saliva pellets was examined, not cell-free saliva, and a set of 3 mRNAs served as controls. The original publication from the Wong group demonstrating mRNAs with increased concentration in saliva associated with OSCC was based on 32 patients with early stage OSCC and 32 healthy, age and smoking history matched controls [29]. Quality control was the detectability of housekeeping RNAs by RT-PCR. Microarray analysis initially showed 17 mRNAs were overrepresented in cell-free saliva of OSCC patients while no genes were downregulated. This may suggest an increased overall level of RNA in saliva of OSCC patients. Remarkably, after focusing on the genes known to be associated with cancer, 7 out of 9 showed increased levels in saliva using RT-PCR; these were IL8, IL1B, DUSP1, HA3, OAZ1, S100P, and SAT. Li et al. then created a classifier for OSCC based on levels of these 7 RNAs in saliva, reporting a sensitivity of 0.91 and a specificity of 0.91 using the same training set [29]. A study from the same group in 2010 focused on a Serbian population differing in race and including all OSCC tumor stages. In that study, 6 out of the 7 original mRNAs were tested using nested RT-PCR, and all values were normalized to control RNAs from 3 housekeeping genes, not to saliva volume as in the first study [41]. While the study did validate the increase in levels of 4 out of 6 RNAs tested in this population of patients with OSCC, it by no means validated their value as a classifier for OSCC as substantial changes were made to the original classifier after testing multiple alternatives. It will be important to validate this new classifier of 4 saliva RNAs and 3 saliva proteins, without modification, in a study of independent subjects. 

 The discovery that extracellular RNA in saliva is protected in exosomes added great strength to the idea that RNA in cell-free saliva could be used to diagnose systemic disease. The Wong group has done multiple studies linking changes in saliva RNA to systemic disease including ovarian and pancreatic cancer [35, 36]. While the two studies named do show differential expression of certain marker RNAs in saliva for both diseases using two sets of patient samples, they did not maintain separate test and validation patient sample sets when developing the classifier predictive of the disease under study. For that reason, it is likely that the high level of sensitivity and specificity for disease identification by measuring the mRNAs they recommend will likely decrease when a true independent set of patient saliva samples is examined and that further refinement of the classifier will be necessary with the measurement of additional or different RNAs. The initial observation of relatively intact RNA in the extracellular saliva by the Wong group was a seminal finding. However, validation for all the disease classifiers with an independent set of subjects is needed before the use of salivary RNA as a screen for disease can be fully evaluated [37–39].

## 4. Epigenetic Changes in DNA from Cells in Saliva

Alterations in gene promoter methylation provide a method to detect OSCC by DNA analysis. Hypermethylation of CpG islands in the promoter regions of many tumor suppressor genes has been linked to the loss of expression of these genes that occurs with the multiple tumor types that make up the family of HNSCCs. In recent years, this method of tumor cell analysis has overshadowed mutational analysis of the HNSCC or OSCC genome as having promise to identify these diseases. To perform methylation analysis, DNA isolated from tumor tissue is exposed to bisulfite to convert cytosines not methylated at the 5 position to uracil, followed by hybridization of oligonucleotide primers to the altered sequence and detection using PCR. This produces a quantitative measure of methylation at specific CpG sites after normalization to a housekeeping gene promoter site known not to show differential methylation. While these analyses have been done with surgically obtained oral tissue, they can also be done with exfoliated cells from saliva or an oral rinse with or without a previous brushing to dislodge cells [42, 43]. This greatly simplifies cell acquisition, especially from unseen pharyngeal lesions, though it dilutes the numbers of cells that come directly from the lesion. Possibly because tumors may tend to shed cells at high rates and field cancerization can cause changes in gene expression in wide areas of tissue around a tumor, the method seems to work. Usage of exfoliated cells and not surgically acquired tumor tissue showed only a slight decrease in sensitivity to promoter methylation detection and then only for some genes [44, 45].

A major stumbling block to DNA methylation studies in the past was the relatively large amount of material needed to perform the analysis and the lack of a true high throughput approach to catalog differential promoter methylation. While DNA microarray hybridization analysis allows the measurement of the levels of thousands of RNAs in one experiment, methylation studies of CpG islands in promoters have, until recently, been done one gene at a time, typically on genes first identified as hypermethylated in HNSCC cells lines. As one might expect hyper- and hypo-CpG methylation patterns that occur in HNSCC cell lines can be markedly different than those seen in tumor tissue [46]. Of the published records, of about 50 prospective genes tested as methylation markers for HNSCC or OSCC in saliva, only a few have shown utility to accurately identify these diseases [44]. Nevertheless, this approach has provided studies linking hypermethylation of certain genes in cells found in oral rinses to not only HNSCC but also moderate-to-severe dysplasias based on EDNRB promoter methylation and to verrucous HNSCC relapse prediction based on methylation status of specific promoter sites in a panel of 5 genes [45, 47].

 In the last few years, high throughput DNA methylation assays have become available that offer multiplexed systems for determination of CpG methylation status at thousands of sites at once [44, 48]. As an example, recent work using this approach with oral tissue from 4 OSCCs, 4 leukoplakia patients, and 4 healthy controls detected 301 hypermethylated and 62 hypomethylated genes promoter sites [44]. That research focused on 8 genes shown to be underexpressed in HNSCC and also hypermethylated in at least some tumor types in earlier studies. Four of these were used to identify SCC among a validation set of 55 HNSCC and 37 normal surgically obtained tissue samples, with 94% sensitivity and 97% specificity. Accuracy dropped greatly when used on DNA harvested from oral rinses of patients with HNSCC and controls. The loss of accuracy may be due to the use of saliva cells while the classifier was developed with tumor tissue. As noted, certain genes show poor correlation in methylation status between tissue and exfoliated cells. This suggests that, if possible, exfoliated cells from patients should be used directly for the multiplexed analysis of gene methylation status with HNSCC though the amounts of DNA are more limited. With the ability to rapidly screen many methylation sites, DNA hypermethylation analysis of oral rinse cells may allow the rapid identification of genes ideal for OSCC and oropharyngeal cancer classification. It also must be recognized that OSCC and oropharyngeal cancer can be different cancers with different etiologies, gene expression, and mutation patterns and that attempts to provide a single classifier for all the forms may be difficult or impossible [49, 50].

Methylation-based HNSCC diagnosis using saliva cells holds much promise. DNA is much more stable than RNA, and it is assumed that even in exfoliated cells the loss of methylation and DNA degradation occur at low rates and are not a factor, though that will need to be verified. As has been noted, standardization of sample acquisition and DNA purification will be necessary [48]. It will be interesting to see if saliva DNA methylation analysis will show sufficient sensitivity to detect oral premalignancy or if it will be necessary to use a cytology brush to increase the number of cells from the lesion. It will be crucial to show that the methodology not only differentiates HNSCC from healthy mucosa but also from benign pathology, such as reactive inflammation, to increase specificity in the clinic. Examination of oral cells also may prove useful in the analysis of some systemic diseases. Methylation of gene promoters of cells in saliva or obtained with a brush from the oral mucosa has been linked to cigarette smoke-induced changes in the lungs [51–53]. It has been suggested as an alternative to lung biopsy to noninvasively size up changes in the lungs using saliva DNA methylation analysis with the goal of providing guidance on lung cancer susceptibility. Furthermore, the characterization of cells in sputum from the respiratory system showed changes in DNA promoter methylation associated with lung cancer [54]. It is speculated that traces of DNA in respired air can also be scanned for the presence of methylation of specific sites of the DNA to track lung cancer, though this approach is in its infancy [55].

## 5. Discussion

RNA from brush cytology and DNA from oral rinses come almost exclusively from epithelial cells of oral and adjoining tissue mucosa. As stated earlier homogeneous samples offer more sensitive detection of RNA population changes in the cells of interest. It may also eliminate much of the variability between studies that use surgical samples harvested in different ways and thus containing variable amounts of stroma, a problem that has plagued between-laboratory comparisons of gene expression-based classifiers. While studies using oral cells to measure RNA, or DNA methylation, have taken advantage of genes identified in earlier studies of surgically obtained tissue, new genes better suited to OSCC and HNSCC identification may also come to light, given the pure cell population used. 

 Analysis of RNA from brush cytology and methylated DNA from oral rinses shows great promise to offer screening for oral disease. There is a need for more evidence that both methods work with external validation sets, though less so for analysis of DNA from oral rinses where more studies have been done. While studies on RNA from brush cytology has compared samples from malignant versus benign lesions, most research on DNA methylation in oral rinses have compared malignant versus healthy tissue. Thus there is a need to establish if the latter method can differentiate malignant versus benign disease. 

RNA from cell-free saliva may allow detection of oral and systemic disease based on changes in RNA in the saliva. This is thought to be due to the presence of RNA released throughout the body and carried to the oral cavity via the blood. The high number of cell types thought to be contributing to the extracellular saliva RNA population makes it difficult to suggest there will be RNA signatures sufficiently specific to systemic diseases to allow accurate detection of a large number of these diseases. However, the possibility of using this approach to detect oral and oropharyngeal disease where the diseased cells should be a dominant RNA source is promising for this method whose great virtue may be the simplicity of sample collection. Interestingly, recent analyses from several groups suggest that the analysis of miRNA in both unfractionated and cell-free saliva may have potential to provide a classifier for OSCC [56–58].

 A major question is how will any of the three methodologies be used in the clinic if they do prove to add value to diagnosis? There is great commercial interest in the development and manufacture of portable instrumentation for automated isolation of DNA and RNA from samples outside the laboratory, performance of cDNA synthesis, or bisulfite reaction, followed by PCR allowing quantification of the sequences of interest. Direct hybridization detection of targets will in time simplify the methodology. While these machines are chiefly designed for use with blood, they are adaptable to saliva analysis and should provide point-of-care rapid diagnosis of oral disease in the dental or medical clinic, eliminating the need for patient referral for this in just a few years.

## 6. Conclusion

Noninvasive methods using gene expression analysis to detect early oral malignancy will likely be used in the clinic to provide point-of-care detection in five years time and for definitive diagnosis sometime after that. At this point, the analysis of DNA methylation has the most promise to be the method of choice. Unlike RNA from brush cytology, DNA methylation has had a large number of resources directed toward it, and, differing from analysis of extracellular RNA in saliva, it has shown utility by several research groups. Recent findings provide encouragement that the days of missing early and curable cancerous lesions of the mouth and pharynx for lack of a simply applied screen are coming to a close.
